# Quantum Sensing of Free Radical Generation in Mitochondria of Human Keratinocytes during UVB Exposure

**DOI:** 10.1021/acssensors.4c00118

**Published:** 2024-05-14

**Authors:** Siyu Fan, Lluna Lopez Llorens, Felipe P Perona Martinez, Romana Schirhagl

**Affiliations:** Department of Biomaterials & Biomedical Technology, University Medical Center Groningen, University Groningen, Antonius Deusinglaan 1, 9713 AV Groningen, The Netherlands

**Keywords:** nanodiamonds, diamonds, skin cells, UV irradiation, NV centers

## Abstract

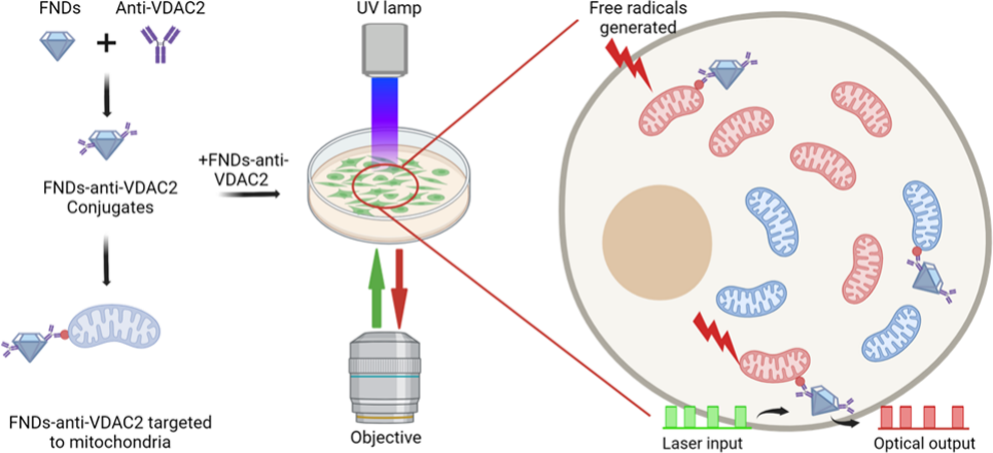

Ultraviolet (UV) radiation is known to cause skin issues, such as dryness, aging, and even cancer. Among UV rays, UVB stands out for its ability to trigger problems within cells, including mitochondrial dysfunction, oxidative stress, and DNA damage. Free radicals are implicated in these cellular responses, but they are challenging to measure due to their short lifetime and limited diffusion range. In our study, we used a quantum sensing technique (T1 relaxometry) involving fluorescent nanodiamonds (FNDs) that change their optical properties in response to magnetic noise. This allowed us to monitor the free radical presence in real time. To measure radicals near mitochondria, we coated FNDs with antibodies, targeting mitochondrial protein voltage-dependent anion channel 2 (anti-VDAC2). Our findings revealed a dynamic rise in radical levels on the mitochondrial membrane as cells were exposed to UVB (3 J/cm^2^), with a significant increase observed after 17 min.

The skin serves as a vital protective barrier, which is crucial for maintaining internal balance in organisms. However, exposure to solar ultraviolet (UV) rays can compromise the integrity of the skin barrier.^1,2^ Among these rays, UVB radiation (260–320 nm) poses a significant threat to the skin, leading to various injuries characterized by inflammatory and repair reactions, as well as the generation of free radicals and apoptosis. These effects manifest in immediate consequences like erythema and hyperpigmentation.^3−5^

It is important to note that UVB radiation mostly affects the epidermis, the outermost layer of the skin mainly composed of keratinocytes, as it does not penetrate deeply.^4^ Free radicals, specifically the superoxide radical (O_2_^•–^) and hydroxyl radical (HO^•^), play crucial roles in regulating the responses of skin cells to UVB exposure. These radicals have been implicated in processes, such as skin inflammation and cancer.^6,7^

The significance of radicals in UVB-induced damage to keratinocytes is underscored by evidence, showing that exposure to both enzymatic and nonenzymatic antioxidants reduces UVB-induced apoptosis.^8−10^ This emphasizes the intricate relationship among UVB radiation, free radicals, and their impact on skin health.

Mitochondria play a major role as a primary source of radicals in UVB-irradiated keratinocytes. UVB exposure disrupts mitochondrial electron transport, leading to a decline in oxygen uptake, ADP phosphorylation, and mitochondrial membrane potential (ΔψM).^3,7^ Simultaneously, it triggers an increase in radical production due to incomplete reduction of oxygen.^4^ UVB irradiation also induces the release of cytochrome c from mitochondria, activating caspases and initiating apoptosis.^11,12^ The impact of UV radiation on mitochondrial physiology has been studied in various cell types.^13−17^ However, the real-time kinetics of free radicals in mitochondria, especially in keratinocytes, remains incompletely characterized due to the instability and reactivity of free radicals.^2,4,16^

Current methods for measuring free radicals, often relying on fluorescent dyes, suffer from bleaching over time. Additionally, they provide historical (from radicals that were present between adding the dye and the measurement) rather than real-time data. In this study, we addressed these challenges by employing nonbleaching fluorescent nanodiamonds.^18^ FNDs contained nitrogen-vacancy (NV) centers that possess the ability to sense unpaired electrons in free radicals at the nanoscale. These NV centers alter their optical properties based on the surrounding magnetic environment. Due to the ease of measuring optical signals compared to small magnetic signals, this technique is highly sensitive.^19^ NV centers have proven successful in various nanoscale sensing applications in physics, such as measuring magnetic nanostructures, nanoparticles, paramagnetic ions, or spin defects.^20−24^ They can also be used for measurements under extreme pressures or temperatures.^25,26^

In the realm of biology, NV centers in diamonds have demonstrated their potential by visualizing spin labels in cell slices,^27^ measuring iron-containing protein,^28^ and enabling nanoscale temperature^25,29^ and orientation measurements.^30,31^ This technique has recently been applied to achieve nanoscale-resolution measurements of free radicals in various biological systems, including aging yeast cells,^32^ immune cells,^33,34^ and endothelial cells^35^ during viral infection^36^ or sperm cell maturation.^37^

In this research, NV centers inside FNDs were utilized to detect spin noise from free radicals using a home-built magnetometer. Additionally, FNDs were conjugated with the anti-VDAC2 antibody through physical adsorption, facilitating the targeting of FNDs to the mitochondria of human keratinocytes (Figure 1). This approach allowed for the dynamic exploration of how and when mitochondria respond to UVB exposure, offering valuable insights into the real-time tracking of free radicals in this specific cell type.

**Figure 1:**
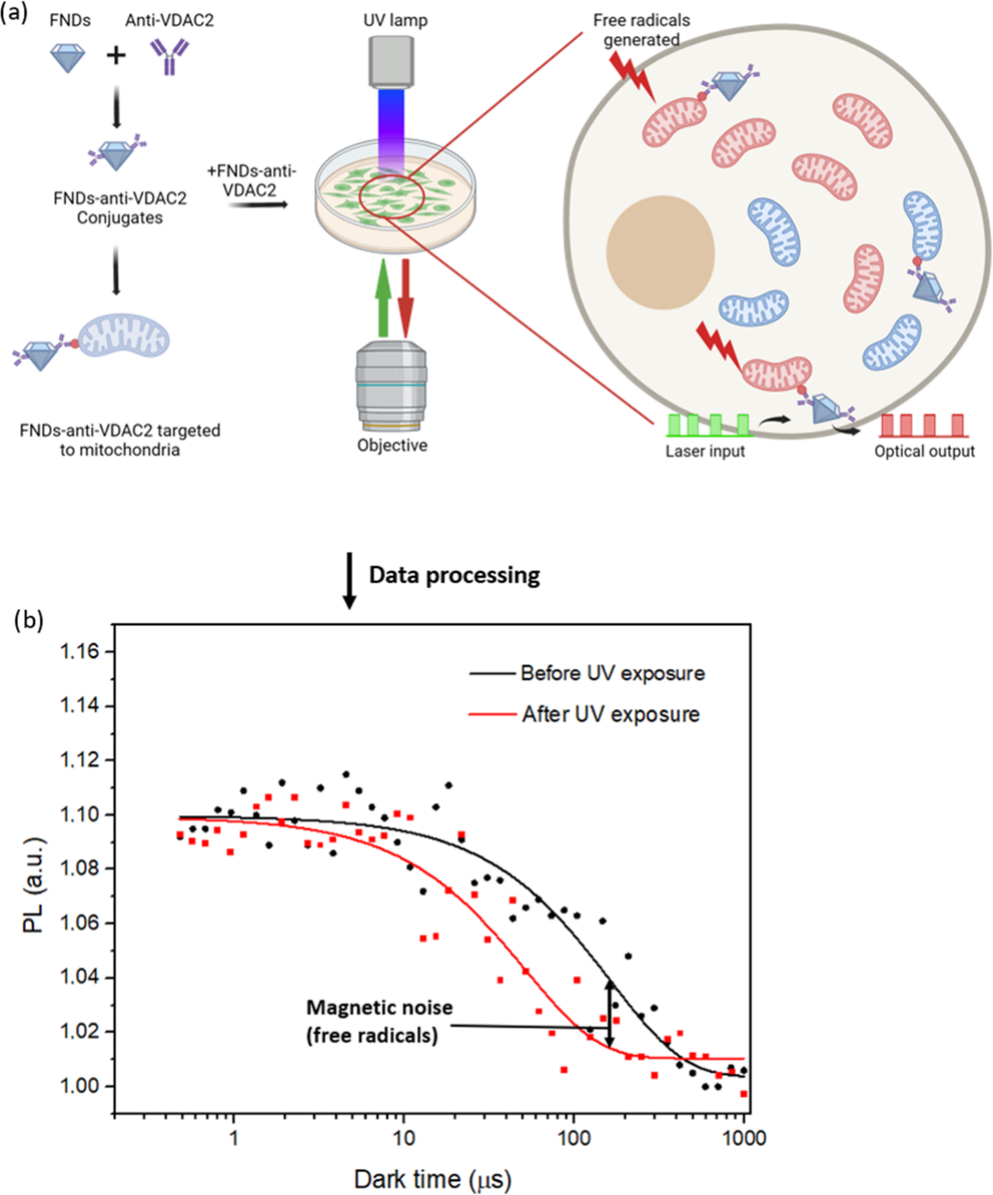
Schematic illustration of the nanodiamond design and T1 relaxometry to sense free radicals near mitochondria in human keratinocytes (HacaT). (a) By conjugating to antibody VDAC2, FNDs are targeted to mitochondria. To do the T1 measurement, NV centers in FNDs are pumped to ms = 0 of the ground state by laser pulses (green blocks), the optical output is then recorded in a certain time window at every beginning of the red blocks (nanodiamonds emission pulses), generating a T1 relaxation curve. Picture is created with BioRender.com (b) Two typical T1 relaxation curves measured in cells when they are exposed to UV (red line) or not (dark line). The time taken to reach equilibrium reflected the quantity of magnetic noise (in this case free radicals) present. Higher levels of free radicals resulted in a quicker decay and a lower T1 value. The UV-exposed group signifies faster relaxation due to magnetic noise in the mitochondrial environment. To ensure precision, the pulsing sequence was repeated 10,000 times for each measurement, ensuring a reliable signal-to-noise ratio.

## Results and Discussion

### Characterization of Fluorescent Nanodiamonds (FNDs) and Particle Uptake

The size distribution of FND and FND-anti-VDAC2 conjugates was determined by dynamic light scattering (DLS) (Figure S1). The average hydrodynamic diameter of FND-anti-VDAC2 (126 nm) increased compared with bare FNDs (88 nm). This dynamic light scattering result aligns with previous findings,^33^ indicating that VDAC2 antibodies can be physically absorbed on the surface of FNDs without requiring additional modifications.

**Figure 2:**
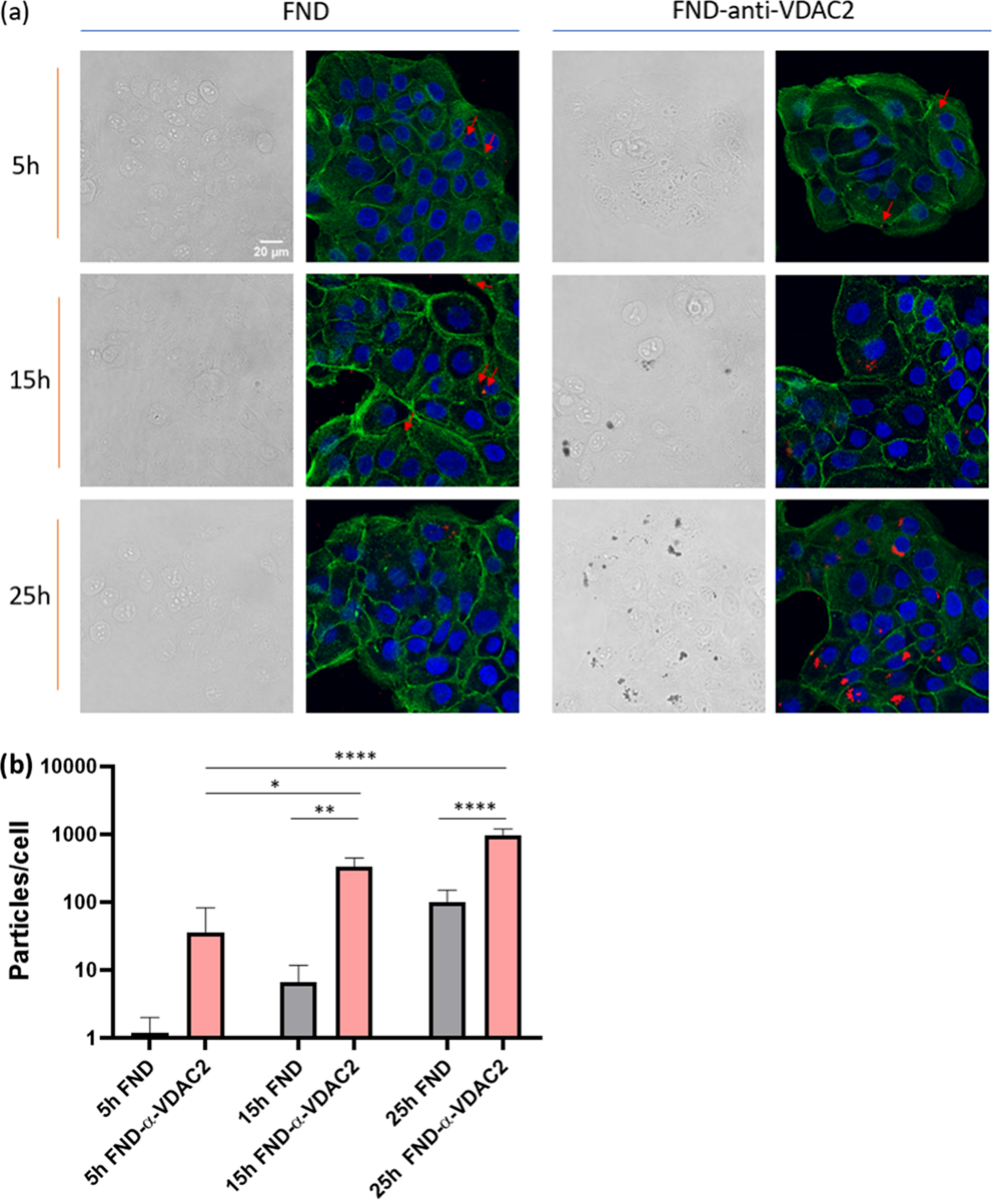
Nanodiamond particle uptake and quantitative analysis in HacaT cells: (a) HacaT cells were incubated with 10 μg/mL FNDs or FND-anti-VDAC2 for 5, 15, or 25 h. Color code: green, phalloidin-FITC, staining actin filaments; blue: DAPI, staining DNA; red: FNDs(-anti-VDAC2). (b) Quantitative analysis of FND uptake per cell after different incubation times. The experiment was independently repeated three times with approximately 60 cells counted in total for each group. Error bars represent standard deviations. Data were analyzed using a two-way ANOVA, and statistical differences are denoted by *p ≤ 0.05, **p ≤ 0.01, and ****p ≤ 0.0001.

To conduct relaxometry experiments near mitochondria, an optimized number of diamond particles must be internalized by the cells. As illustrated in Figure 2a, particle uptake in HacaT cells exhibited a time-dependent increase for both types of FNDs. Specifically, for FND-anti-VDAC2 conjugates, a significant rise in particle quantity was observed after 15 and 25 h of incubation (Figure 2b). Moreover, at 15 and 25 h, the number of ingested particles was markedly higher for the FND-anti-VDAC2 group compared to bare FNDs. VDAC2 was proven to promote clathrin-independent endocytosis.^38^ This unique character might explain the higher cellular uptake of antibody-coated FNDs. In the FND-anti-VDAC2 group, particle aggregation was observed after prolonged incubation (Figure 2a). Slightly aggregated FNDs are recommended for their slowed-down movement speed, which benefits tracking.^34^ However, excessively large particles are not sensitive to magnetic noise (Figure S5). Prolonged incubation times may result in more particles reaching their target, but excessive incubation could lead to proliferation and shifts in the position of FNDs as well. Consequently, we selected a 5 h incubation time as the optimized duration for further experiments.

### Subcellular Location of FND and FND-anti-VDAC2 Conjugates

**Figure 3:**
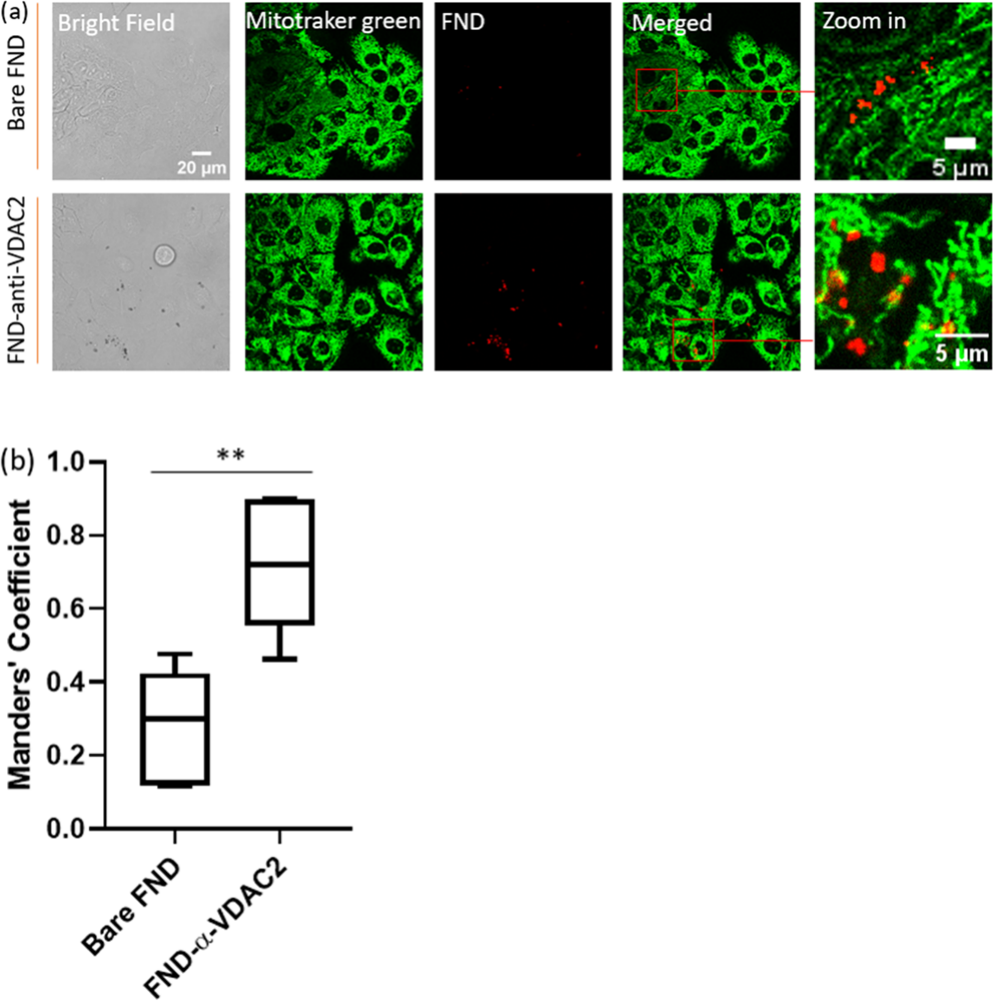
Intracellular location of bare FNDs/FND-anti-VDAC2 in HacaT cells. (a) HacaT cells were incubated with different FNDs for 5 h, and then stained and imaged. Color code: green, MitoTracker; red, FNDs(-anti-VDAC2). (b) Quantitative colocalization analysis of bare FNDs or FND-anti-VDAC2 conjugates and mitochondria after a 5 h incubation. Error bars represent standard deviations. The data were analyzed by one-way ANOVA. **p ≤ 0.01.

To determine where FND-anti-VDAC2 conjugates were located within HacaT cells during T1 measurements following a 5 h incubation, we utilized confocal z-stack imaging (Figure 3a). Mitochondria, marked with MitoTracker Green, showed substantial colocalization with FND-anti-VDAC2 particles, indicating successful targeting after 5 h. Less colocalization was observed with bare FNDs. This was further confirmed by image deconvolution and statistical analysis using FIJI and the JACoP plugin. A notable increase in the Mander’s coefficient was observed in the FND-anti-VDAC2 group compared to bare FNDs (Figure 3b and Supporting Information Table 1), validating our measurement of free radical signals near mitochondria during T1 at the corresponding incubation time.

### Nanodiamond Biocompatibility

To evaluate the biocompatibility of nanodiamonds, we conducted a cell titer assay (Figure S4) on HacaT cells exposed to 10 μg/mL bare FNDs, 10 μg/mL FND-anti-VDAC2, or 5% DMSO for 24 h. DMSO served as a positive control due to its known toxicity. Importantly, we observed no notable difference in cell viability between the control and cells exposed to different FND types, indicating the excellent biocompatibility of FNDs with HacaT cells. These results align with previous literature reporting the favorable biocompatibility of FNDs with various cell types.^39−41^

### Superoxide Detection by Dihydroethidium Assay

**Figure 4:**
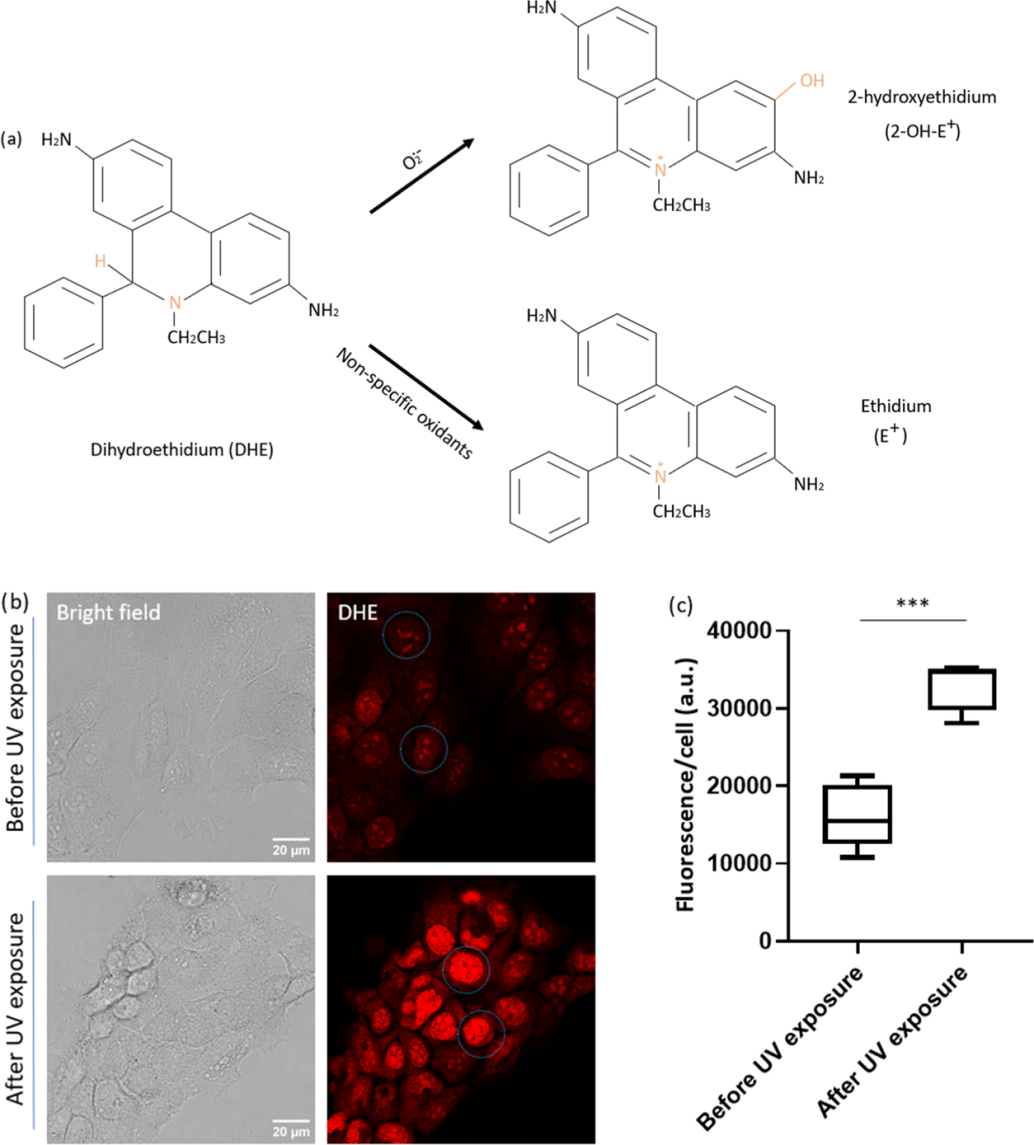
Cellular superoxide measurement in HacaT cells by a DHE assay. (a) The mechanism of DHE oxidation. (b) HacaT cells were first exposed under 0.08 mW/cm^2^ UV for 20 min (weighted exposure to 10 mJ/cm^2^). Hereafter, the DHE was added, and cells were imaged using confocal microscopy. An accumulation of red fluorescence in the nucleus can also be seen (blue circle). (c) Quantitative analysis of average fluorescence signal per cell by FIJI, data between each group were analyzed by an unpaired *t*-test. ***p ≤ 0.001.

The red fluorescence formed from DHE has been used in the detection of intracellular ROS for the last three decades. Oxidants like ONOO^–^-derived oxidants, including ·OH, and higher oxidants derived from peroxidases, react with DHE to form ethidium (E^+^).^42^ The binding of the ethidium cation to polyanions, including DNA, is well-known.^43,44^ However, as mentioned, ethidium is the product of nonspecific oxidation. When DHE enters cells and reacts with O_2_^•–^, the major product of this reaction is 2-hydroxyethidium (2-OH-E^+^). Thus, 2-OH-E^+^ is regarded as a diagnostic marker product of O_2_^•–^(Figure 4a).^45^ To distinguish between 2-OH-E^+^ and E^+^ readouts, specifically focusing on superoxide, which is the major ROS species induced by UV and precursor for other mitochondrial ROS, we employed a selective detection approach for 2-OH-E^+^ using excitation light at 396 nm as recommended.^46^ We observed an enhanced fluorescence intensity in cells (especially in the nucleus area) after 0.08 mW/cm^2^ UV exposure for 20 min (Figure 4b). A significant increase in fluorescence (*p* ≤ 0.001) was further quantified as the average fluorescence intensity per cell (Figure 4c). This observation aligns with other studies, indicating a swift and temporary generation of reactive oxygen species (ROS) in keratinocyte responses to UVB at similar exposing energy.^10,47^

### Free Radical Detection by T1 Relaxometry

While DHE fluorescence is commonly employed for detecting and quantifying ROS levels, it serves as an indirect measure of 2-OH-E^+^ rather than the superoxide itself. The limitations of photostability and spatial resolution further hinder prolonged tracking at specific locations.

T1 relaxometry, based on the sensing of surrounding magnetic noise, can detect the radical response on the surfaces of mitochondria inside the live cell by using FND-anti-VDAC2 conjugates.

**Figure 5:**
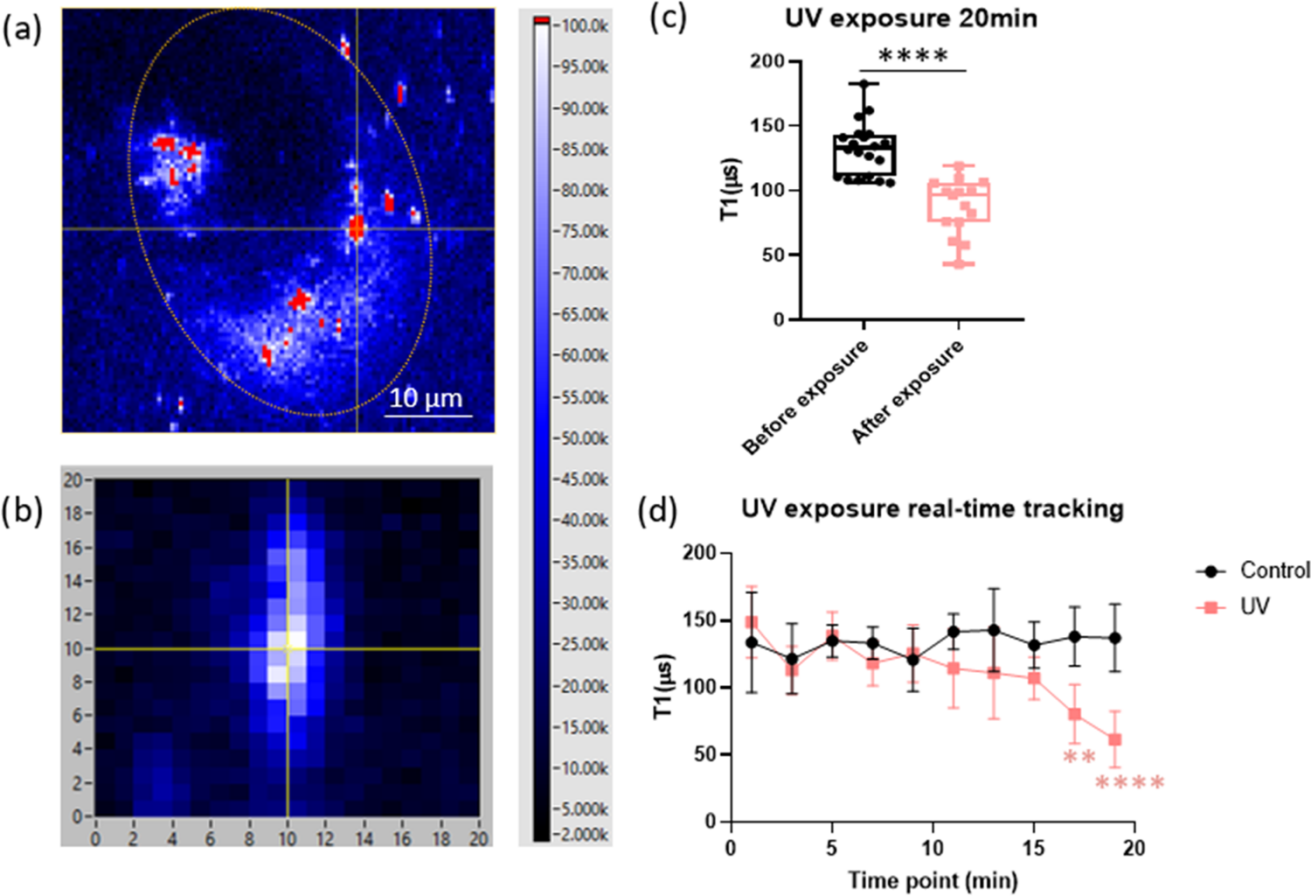
Free radical detection by T1 relaxometry in HacaT cells. (a) Representative fluorescence image of FND-anti-VDAC2 in HacaT cells. The dashed line is the cell border. Intensity bar is shown on the right. (b) Zoom-in of the particle from (a) (the cross point); photon counts are 5 × 10^6^. The particles are tracked during the measurement. An example for a lateral position in *x* and *y* is shown (values are given in μm). Window size is 4 μm × 4 μm. (c) T1 relaxation time of FND-anti-VDAC2 particles in HacaT cells before/after 0.08 mW/cm^2^ UV irradiation for 20 min (weighted exposure to 10 mJ/cm^2^). The results were extracted from the recorded data by biexponential fitting. The experiment was repeated 15 times. (d) T1 real-time tracking obtained from the same FND-anti-VDAC2 particle in UV-irradiated HacaT cells. While UV was switched on, T1 values were recorded every 2 min to observe the dynamic change. Each curve represents an average of 5 measurements, and T1 values of the UV group were statistically compared to the control group for corresponding time points. Significance between groups was analyzed by an unpaired *t*-test (c) or a two-way ANOVA analysis (d). **p ≤ 0.01, ****p ≤ 0.0001.

The setup, detailed earlier,^32^ used the pulse sequence illustrated in Figure 1a to perform relaxometry. For a single measurement, first, baseline T1 was measured. Then, HacaT cells were exposed to 0.08 mW/cm^2^ UV light for 20 min. After exposure, a faster biexponential decay was observed, indicating a higher radical concentration (see Figure 1b). The measurement was then repeated 15 times. The decay velocity of different curves was quantified as the T1 value (a parameter from the mathematic function, which fits the biexponential curve, see eq 1 in the Supporting Information). After comparing 15 T1 values, a significant decrease (****p ≤ 0.0001) was found between the baseline and UV-exposed cells (Figure 5c).

To pinpoint the timing of free radical generation, we continuously monitored near mitochondria during 20 min UVB irradiation. Figure 5d illustrates a gradual decrease in T1 values, signaling an increase in mitochondrial free radical presence over time. Significantly divergent T1 values emerged from 17 min onward.

To assess the UV effect on T1 measurements, a Petri dish (*d* = 35 mm) with dry FNDs attached on the bottom was prepared. For the free radical measurement, we used the same settings as we used in cells. First, the baseline was measured, then the FNDs were continuously irradiated by 0.08 mW/cm^2^ UV irradiation for 20 min (weighted exposure to 10 mJ/cm^2^). T1 relaxation times of 15 measurements were extracted from the recorded data by biexponential fitting. Figure S2 reveals no measurable impact from 0.08 mW/cm^2^ UV irradiation to FNDs.

In cells, T1 measurements using bare FNDs during UV exposure (Figure S3) showed a slight increase (*p* ≤ 0.1) in radical levels, suggesting free radical generation in other cell areas, with the majority still near mitochondria.

Mitochondria usually facilitate the controlled flow of electrons in the electron transport chain, ultimately leading to the formation of water.^48^ However, reduced electron transport chain capacity, often seen in UV-irradiated keratinocytes, can result in the production of superoxide radicals (O_2_^•–^) due to the leakage of single electrons at mitochondrial complexes II and III.^49^ Also, mitochondria contain various UVB-absorbing chromophores, such as amino acids, DNA, and RNA.^50^ When these chromophores absorb UVB radiation, they become excited-state molecules that, upon returning to the ground state, transfer energy to nearby intracellular molecules, particularly oxygen (O_2_). This process leads to the generation of superoxide radicals, which can further dismutate to form nonradical hydrogen peroxide (H_2_O_2_)—a precursor to the highly reactive hydroxyl radical.^2,49^ In the end, this might result in the necrosis of cells^51^ as the intracellular ATP level shows a significant decrease after 20 min UV treatment (Figure S6).

As recently reported, ferroptosis is also activated in the epidermal keratinocytes after their exposure to UVB.^52^ Mitochondria are crucial parts of the ferroptosis process. During this process, iron will accumulate in mitochondria.^53,54^ As iron is paramagnetic in some oxidation stages, it could potentially show some impact on the T1 measurement, leading to a decrease of the T1 value.

## Conclusions

Despite the recognized role of radicals in UVB-irradiated keratinocytes, methodological limitations have hindered the selective measurement of radical levels at the subcellular level in real time.^10,47,55^ In this study, utilizing a radical-specific detection technique, we demonstrate that UVB irradiation distinctly elevates mitochondrial stress in human keratinocytes. While fluorescence-based assays, such as DHE, offer advantages, such as simplified sample processing and simultaneous analysis of numerous samples, they also have drawbacks. These include DHE’s photosensitivity, potential interference from E^+^ absorption and fluorescence, limited spatial resolution, and indirect measurement of 2-OH-E^+^ rather than superoxide itself.

In contrast, T1 measurements provide continuous and real-time information without the limitations associated with traditional assays. Unlike DHE, T1 measurements avoid issues like photosensitivity, potential interference from E^+^, and indirect detection of 2-OH-E^+^.^42^ Moreover, the excitation of the products of DHE oxidation may stimulate further DHE oxidation, as has been shown in the case of 2-OH-E^+^.^56^ T1 measurements specifically capture local information from the mitochondrial surface, confirming the occurrence of free radical generation during UVB exposure in HacaT cells. Additionally, since measurements can be done in sequence, each cell can function as its own control before an intervention. Overall, relaxometry proves to be valuable in enhancing our understanding of oxidative stress responses in UVB-irradiated human keratinocytes.

## Supporting Information Available

Materials and Methods, DLS results for bare and coated FNDs, control measurements for T1 (UV irradiation of particles without cells), control measurements for T1 with bare particles in cells, viability assay results, T1 measurements with large aggregates, cell titer assay before and after UV exposure, confocal images to measure colocalization with lysosomes, Mander’s coefficients for colocalization between FNDs and mitochondria, cellular superoxide measurement in HacaT cells by MitoSOX RED assay (PDF)
