# Supplementary material for: Quantum Sensing of Free Radical Generation in Mitochondria of Human Keratinocytes during UVB Exposure
Source: ACS Sens. 2024 May 14;9(5):2440–6. doi: 10.1021/acssensors.4c00118 (PMC11129351; doi:10.1021/acssensors.4c00118)
Supplement: Supplementary file 1 — se4c00118_si_001.pdf [file se4c00118_si_001.pdf]

Supporting information

Quantum sensing free radical generation in mitochondria of Human Keratinocytes during UVB exposure

Siyu Fan, Lluna Lopez Llorens, Felipe P. Perona Martinez, Romana Schirhagl*

Department of Biomaterials & Biomedical Technology, University Medical Center Groningen, University Groningen, Antonius Deusinglaan 1, 9713 AV Groningen, The Netherlands

Material and methods

Materials

FND particles (Adamas Nanotechnologies, North Carolina, USA) have a hydrodynamic diameter of 70 nm and over 300 nitrogen-vacancy (NV-) centers per particle (manufacturer information). They were synthesized through high-pressure high-temperature synthesis, followed by high-temperature annealing[1]. A cleaning process in oxidizing acid makes the surface oxygen-terminated[2]. This size of FNDs was chosen for their brightness, ease of tracking, and high NV center count, ensuring a favorable signal-to-noise ratio. Opting for even larger particles was deemed less ideal, as larger particles would result in NV centers being too distant from the diamond surface, impacting the effectiveness of the measurements. Each measurement in this study provided an average of all nitrogen-vacancy (NV) centers in a particle, contributing to the increased reliability of the signals from the particles. The biocompatible FNDs maintained stable fluorescence after cellular uptake, contributing to their suitability for biological applications[3,4].

Diamond preparation

Antibody conjuagtion was established in prior research[5]. In this study, 0.1 mg/mL anti-VDAC2 (GeneTex, The Netherlands) were mixed with 10 μg/mL FNDs at a 1:4 ratio, vortexing for 1 minutes, then incubated at room temperature for 10 minutes, resulting in the formation of FND-anti-VDAC2. Changes in size following nanodiamond modification were assessed using the Malvern ZetaSizer Nanosystem (Malvern Instruments Ltd., UK).

Cell culture

Human keratinocytes (HaCaT) cells (CLS, Eppelheim, Germany) were grown in DMEM supplemented with 10% FBS and 100 U/ml of penicillin and streptomycin at 37°C and 5% CO_2.

FND uptake in HacaT cells

For FND uptake analysis, cells (50,000 cells/mL) were seeded in 35 mm glass bottom Petri dishes and incubated with bare FNDs or FND-anti-VDAC2 (10 μg/mL) for 5h, 15h or 25h at 37°C with 5% CO_2. After incubation, FND-containing medium was removed, and cells were washed with 1× PBS, fixed with 3.7% formaldehyde, and stained with DAPI and FITC-phalloidin. Z-stack confocal images were taken with a Leica SP8x microscope (Leica, Germany). FNDs were detected at 561/659 nm, and DAPI and FITC were imaged at 358/461 nm and 495/510 nm, respectively.

Z-stack confocal images capturing the entire cell volume were obtained. Approximately 60 randomly selected cells from each of the three independent experiments were analyzed using the 3D object counter plugin of FIJI, setting a size filter of 8 pixels and a gray level threshold

of 20. This threshold was determined as the lowest possible value where the control group signal was zero.

Subcellular location of FNDs in HacaT cells

To determine the intracellular location of diamond particles within HacaT cells at different incubation times, Mito Tracker Green (Gibco, Thermo Fisher Scientific, The Netherlands) was employed to label mitochondria. Cells were seeded at a density of 50,000 cells/mL in 35 mm glass bottom Petri dishes and incubated with 10 µg/mL of bare FNDs or FND-anti-VDAC2 for 5 hours. Subsequently, cells were washed with PBS to prevent continuous FND uptake, enabling the tracking of previously endocytosed FNDs. Mito Tracker Green at a final concentration of 1 µg/mL was added, and cells were incubated for 30 minutes. Live cell imaging was performed using a SP8x Leica confocal microscope.

Approximately 60 random cells from each of three independent experiments were selected for analysis. FNDs were detected at ex/em = 561/659 nm, and Mito Tracker Green was imaged at 495/510 nm. To enhance the signal-to-noise ratio, z-stack images were deconvoluted using the Diffraction PSF 3D and iterative deconvolve 3D plugins in FIJI. Subsequently, the JAcoP plugin (https://imagej.nih.gov/ij/plugins/track/jacop.html) in FIJI was employed to determine whether FNDs colocalized with Mitotracker Green. The Manders' Coefficient (MC), a widely used metric for organelle colocalization analysis, indicated the fraction of FNDs within compartments containing mitochondria[6].

Cell viability test

To assess cell viability, the CellTiter-Glo Luminescent Cell Viability Assay from Promega was employed. This assay measures ATP levels as an indicator of metabolically active cells. HacaT cells were seeded in clear flat-bottom 96-well plates at a density of 50,000 cells per well. Following the removal of the cell culture medium, cells were rinsed once with phosphate-buffered saline (PBS). Subsequently, the cells were incubated with either 10 µg/mL FNDs/FND-anti-VDAC2 or 5% Dimethyl sulfoxide (DMSO) as a positive control for 24 hours. After the incubation period, the plate and its contents were equilibrated to room temperature for approximately 30 minutes. Then we added 100 µL of CellTiter-Glo 2.0 Reagent to 100 µL of medium-containing cells, with thorough mixing for 2 minutes on an orbital shaker. The plate was then incubated at room temperature for 10 minutes to stabilize the luminescent signal. Luminescence was measured using a FLUOstar Omega Microplate Reader (BMG Labtech, located in De Meern, The Netherlands), with untreated cells serving as a negative control.

UV exposure

Before UVB irradiation, cells were washed three times with warm phosphate-buffered saline and re-fed with their own medium. Simultaneously to the relaxometry measurements, the HaCaT cells were irradiated with UVB light (peak emission at 275 nm) at an irradiance of ~0.08 mW/cm^2 (Thorlabs Mounted LED, M275L4). Each measurement lasted 20 minutes, accumulating a fluence of ~10 mJ/cm^2. The UVB intensity was measured using a Thorlabs PM100D power meter with an S130C photodiode at the position of the sample.

Superoxide detection by Dihydroethidium (DHE) assay

HacaT cells were seeded in a 35mm Petri dish with a density 50,000 cells/mL. After a 20-minute UVB exposure, the cells were washed with PBS. Following this, a solution of DHE (2

μg/mL) in DMEM medium was added to the cells. This reagent is specifically used for detecting reactive oxygen species (ROS) like intracellular superoxide. The cells were then incubated for 10 minutes at 37°C with 5% CO_2. Subsequently, the fluorescence intensity, indicative of ROS generation, was measured using a Leica Sp8x confocal microscope at excitation and emission wavelengths of 396 nm and 550-600 nm, respectively. This wavelength is used to selectively measure superoxide[7]. Negative controls consisted of non-treated cells. Confocal images were subjected to analysis using the FIJI software (http://fiji.sc/) to quantify the average fluorescent intensity across approximately 60 randomly selected cells. Integrated density (Int Den) was employed, representing the sum of pixel values within the image or selection per cell, indicating the level of superoxide. A control group was utilized to subtract background values from other experimental groups.

Free radical measurements in HacaT cells by T1 measurement

A previously described home-built magnetometry setup was employed for T1 measurements[8]. The setup is essentially a confocal microscope with an acousto-optical modulator (Gooch & Housego, model 3350-199) for detection. Glass bottom Petri dishes with HacaT cells were incubated overnight. To explore free radical generation near mitochondria during UVB exposure, HacaT cells were treated with 10 μg/mL FND-anti-VDAC2 for 5h, followed by PBS washing and UVB exposure for 20 minutes. T1 measurements were immediately performed and repeated 15 times for each group, with non-treated cells as controls. For real-time tracking, T1 measurements were conducted on the same particle within a HacaT cell and continuously monitored for 20 minutes to track free radical generation. This real-time tracking experiment was repeated five times.

In T1 relaxometry measurements, NV defects in diamonds were employed for quantum sensing at room temperature. The NV centers in diamonds optically read the magnetic noise of the surrounding region[2]. Using the pulse sequence illustrated in ***Figure 1***a, relaxometry was performed, correlating the decay velocity with magnetic noise arising from free radicals.

In a standard T1 measurement, NV centers were brought into the bright ms=0 state, probing after different times to see if the NV centers remained in this state or returned to the equilibrium between ms=0 and ms=+1 and -1.

For pulsing, the NV centers were excited with a series of 5 μs green laser pulses (561 nm) with a dark time (τ) ranging from 200 ns to 10 ms. To generate relaxometry curves, the brightness in the first 0.6 μs of each pulse was plotted against the dark time (illustrated in ***Figure 1***b). From these curves, T1 was calculated using a bi-exponential model involving a short T_S and a long T_L component (equation shown below), where T_L represents final T1 value.

$$I(\tau) = I_\infty \left(1 + C_S e^{-\tau/T_s} + C_L e^{-\tau/T_L}\right)$$

Equation 1

The photoluminescence intensity at long dark times (τ), denoted as I_∞, represents the final thermal equilibrium. C_S and C_L refer to the short and long contrast of the relaxation curves, respectively, within the bi-exponential model, as elaborated in prior work[9,10]. The time required to reach the equilibrium condition shortened in the presence of free radicals. The pulsing sequence was repeated 10,000 times for each measurement to ensure a satisfactory signal-to-noise ratio. The laser power at the sample location, measured during continuous

illumination, was set at 50 μW. This laser power was chosen to minimize cell damage while being sufficiently high to polarize the NV centers.

Statistical analysis

Data analysis was carried out using GraphPad Prism version 8.0. The significance of the results was assessed employing either a T-test, one-way or two-way ANOVA test (Tukey multiple comparisons), depending on the nature of the specific experiment. Significance was determined by comparing the experimental groups to the control group. The defined significance levels were as follows: ns (not significant) for $p > 0.05$, * for $p \leq 0.05$, ** for $p \leq 0.01$, *** for $p \leq 0.001$, and **** for $p \leq 0.0001$

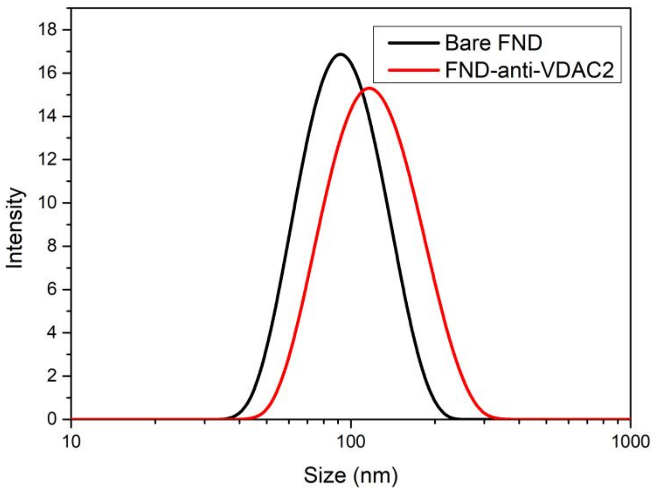

Figure S1. Size measurement of bare FNDs and FND-anti-VDAC2, measured by the Malvern ZetaSizer Nano system. uncoated FNDs (black) exhibited a size of 88 nm (polydispersity index (PDI) = 0.078). FND-anti-VDAC2 (red) measured 126 nm in size (PDI = 0.104). The observed increase in particle size suggests that bare FNDs were effectively coated by antibody.

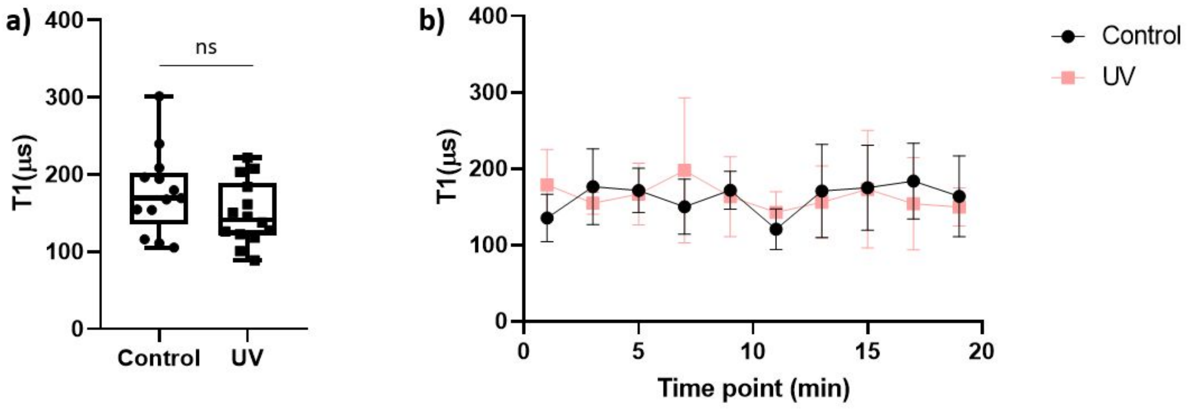

Figure S2. Free radical detection by T1 relaxometry using bare FNDs. a) FNDs were irradiated before/after 0.08mW/cm^2 UV irradiation for 20min (weighted exposure to 10mJ/cm^2). T1 relaxation time of 15 times measurements was extracted from the recorded data by biexponential fitting. b) T1 real-time tracking obtained from the same FND particle. T1 values were recorded every 2 mins. Each curve represents the average of 5 measurements. Data between each group were analyzed by an unpaired t test (a) or a two-way ANOVA analysis (b),ns=no significant difference.

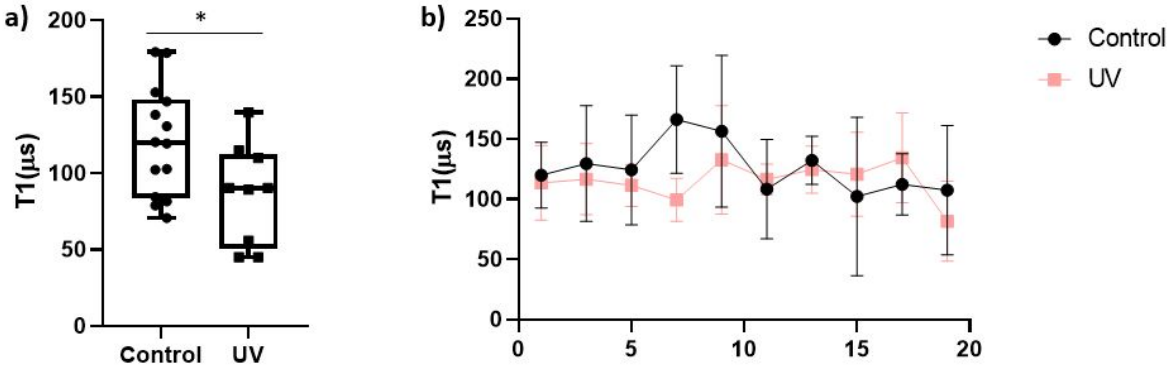

Figure S3 Free radical detection in HacaT cells by T1 relaxometry using bare FNDs. a) T1 relaxation time of 15 times measurements was extracted from the recorded data by biexponential fitting. b) T1 real-time tracking obtained from the same FND particle in UV-irradiated HacaT cells. T1 values were recorded every 2 mins. Each curve represents the average of 5 measurements. Data between each group were analyzed by an unpaired t test (a) or a two-way ANOVA analysis (b), *$p \leq 0.1$.

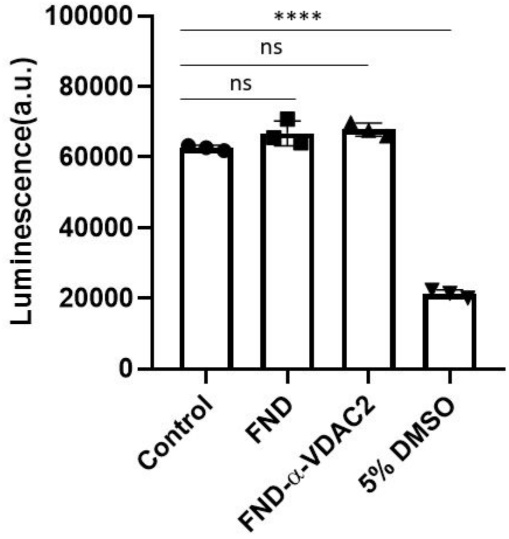

Figure S4. Cell viability was assessed through a Cell Titer assay, repeated thrice for diverse cells. Error bars denote standard deviations, and statistical analysis, performed using one-way ANOVA, revealed significance (****$p \leq 0.0001$, ns= no significant difference).

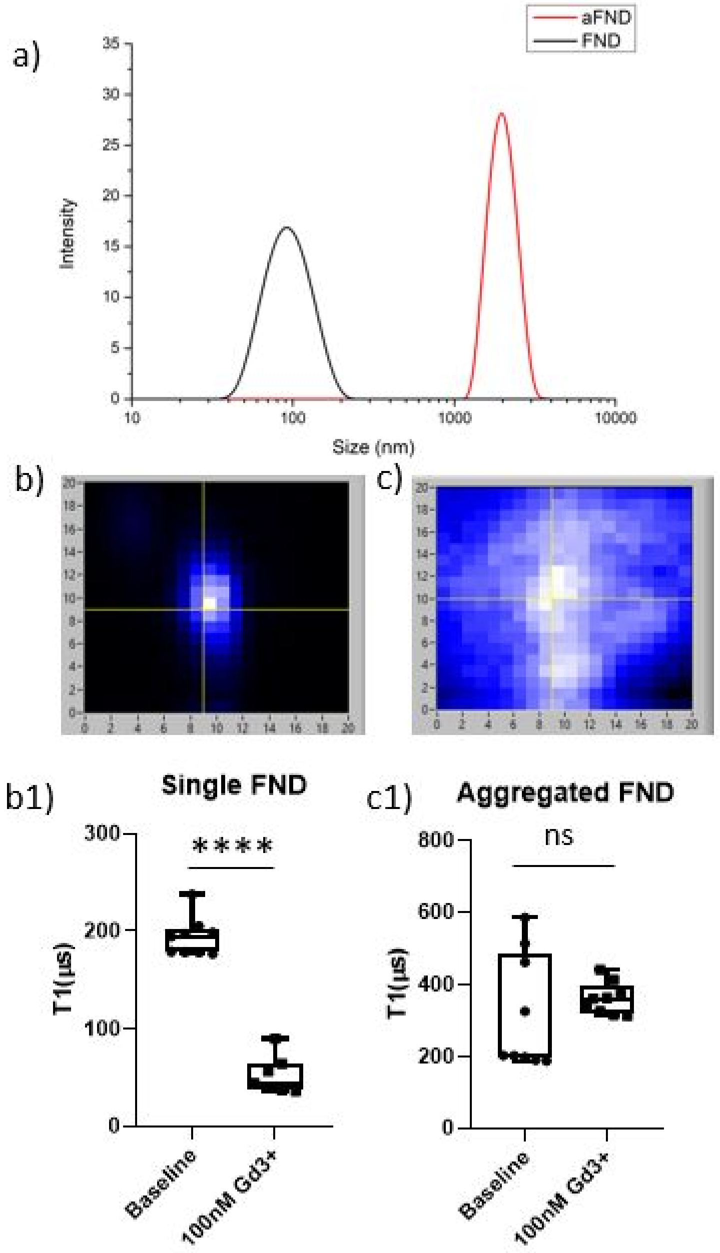

Figure S5. a) Size measurement of single FNDs in water and aggregated FNDs in PBS, measured by the Malvern ZetaSizer Nano system. uncoated FNDs (black) exhibited a size of 88 nm (polydispersity index (PDI) = 0.078). FND-anti-VDAC2 (red) measured 2 µm in size (PDI = 0.118). Particles with different size can be visualized in the Particle-centering window in T1 set-up. b) A single FND particle; c) An aggregated FND particle. Window size is 4µm x 4µm. After centering the FND, the T1 measurement was then performed. b1) T1 measurement of single/aggregated FNDs with/without 100 nM $GdCl_3$. c1) T1 measurement of aggregated FNDs with/without 100 nM $GdCl_3$. FNDs were firstly dried on a Petri dish, then $GdCl_3$ solution (offer magnetic noise) was added to the dish. As can be seen, when FNDs form big aggregation (2 µm), they are not as sensitive as they are in nanoscale size anymore. Significance between groups was analyzed by an unpaired t test. ****p ≤0.0001, ns= no significant difference.

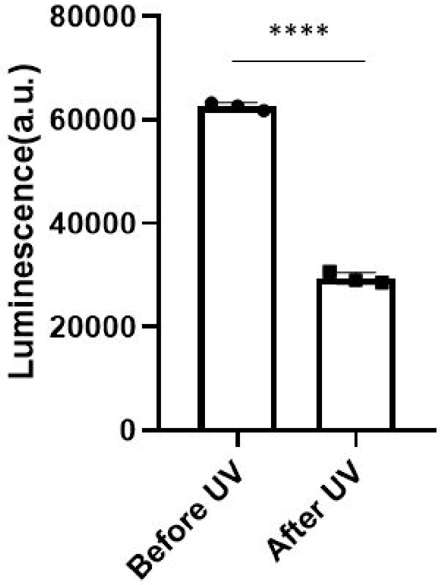

Figure S6. In HacaT cell, before and after 20 mins UV treatment, ATP level was assessed through a Cell Titer assay, repeated thrice for diverse cells. Error bars denote standard deviations, statistical analysis was performed using unpaired t test, significance ****p ≤ 0.0001.

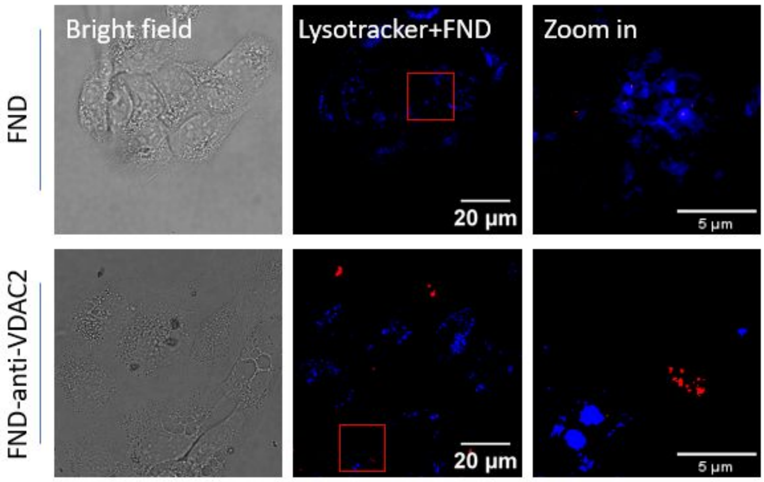

Figure S7. Intracellular location of bare FNDs/FND-anti-VDAC2 in HacaT cells. HacaT cells were incubated with different FNDs for 5h, then stained and imaged. Color code: blue, lysotracker; red, FNDs(-anti-VDAC2). Compared with the FND group, there is barely FND-anti-VDAC2 colocalized with lysosomes.

Table 1 Manders' coefficient of mitochondria and FND/FND-anti-VDAC2, or lysosome and FND/FND-anti-VDAC2 after 5h incubation time (from Figure 3b and Figure S7). The standard deviations are indicated in the table. The data were analyzed by a two-way ANOVA. For FND-anti-VDAC2, a significant difference (**p ≤0.01) of colocalization was found between mitotracker and lysotracker, which indicated most of the particles were escaped and targeted to mitochondria after 5h.

	MCs
**Bare FND and mitotraker**	0.278±0.158
**FND-anti-VDAC2 and mitotracker**	0.724±0.184
**Bare FND and lysotraker**	0.53±0.34
**FND-anti-VDAC2 and lysotracker**	0.08±0.07

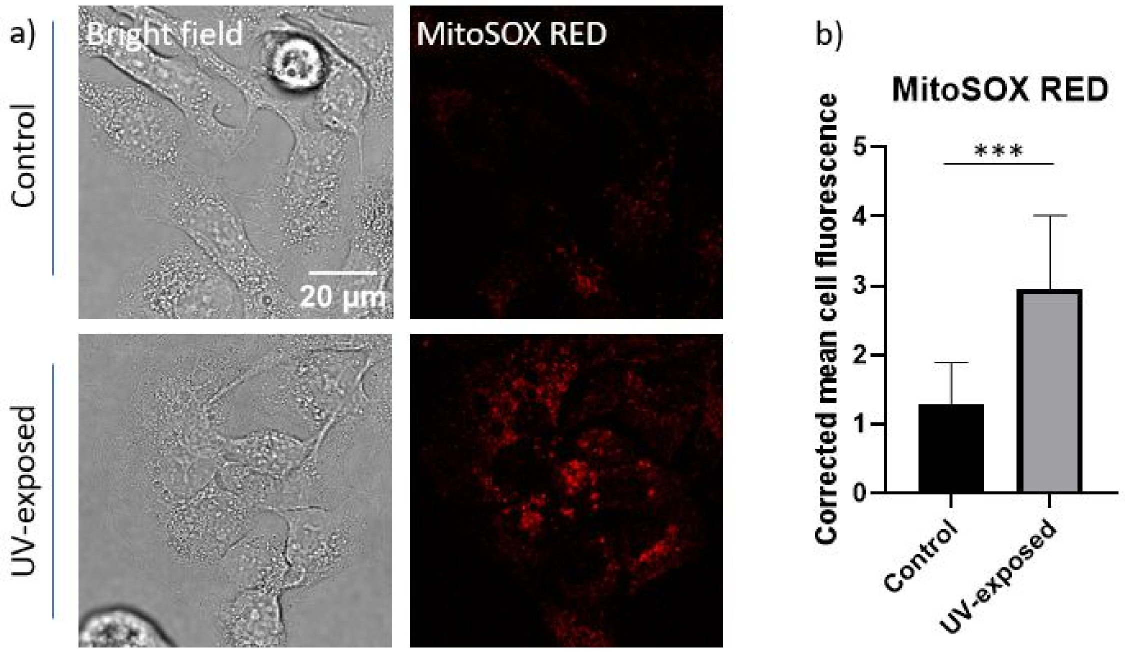

Figure S8. Cellular superoxide measurement in HacaT cells by MitoSOX RED assay. a) representative confocal image of control HacaT cells or UV-exposed cells (weighted exposure to 10mJ/cm^2. More production of superoxide can be seen in UV-exposed group. b) Quantitative analysis of corrected average fluorescent signal per cell by FIJI, around 30 cells were analysed in total. Data between each group were analyzed by an unpaired t test. *** for $p \leq 0.001$.

References

(1) Shenderova, O. A.; Shames, A. I.; Nunn, N. A.; Torelli, M. D.; Vlasov, I.; Zaitsev, A. Review Article: Synthesis, Properties, and Applications of Fluorescent Diamond Particles. Journal of Vacuum Science and Technology. B, Nanotechnology & Microelectronics 2019, 37 (3), 030802. https://doi.org/10.1116/1.5089898.

(2) Schirhagl, R.; Chang, K.; Loretz, M.; Degen, C. L. Nitrogen-Vacancy Centers in Diamond: Nanoscale Sensors for Physics and Biology. 2014. https://doi.org/10.1146/annurev-physchem-040513-103659.

(3) Mochalin, V. N.; Shenderova, O.; Ho, D.; Gogotsi, Y. The Properties and Applications of Nanodiamonds. 2012. https://doi.org/10.1038/NNANO.2011.209.

(4) Mohan, N.; Chen, C.-S.; Hsieh, H.-H.; Wu, Y.-C.; Chang, H.-C. In Vivo Imaging and Toxicity Assessments of Fluorescent Nanodiamonds in Caenorhabditis Elegans. 2010. https://doi.org/10.1021/nl1021909.

(5) Nie, L.; Nusantara, A. C.; Damle, V. G.; Sharmin, R.; Evans, E. P. P.; Hemelaar, S. R.; van der Laan, K. J.; Li, R.; Perona Martinez, F. P.; Vedelaar, T.; Chipaux, M.; Schirhagl, R. Quantum Monitoring of Cellular Metabolic Activities in Single Mitochondria. Sci Adv 2021, 7 (21), 573. https://doi.org/10.1126/SCIADV.ABF0573/SUPPL_FILE/ABF0573_SM.PDF.

(6) Dunn, K. W.; Kamocka, M. M.; McDonald, J. H. A Practical Guide to Evaluating Colocalization in Biological Microscopy. Am J Physiol Cell Physiol 2011, 300 (4), C723. https://doi.org/10.1152/AJPCELL.00462.2010.

(7) Robinson, K. M.; Janes, M. S.; Pehar, M.; Monette, J. S.; Ross, M. F.; Hagen, T. M.; Murphy, M. P.; Beckman, J. S. Selective Fluorescent Imaging of Superoxide in Vivo Using Ethidium-Based Probes; 2006; Vol. 103. www.pnas.orgcgidoi10.1073pnas.0601945103.

(8) Loretz, M.; Pezzagna, S.; Meijer, J.; Degen, C. L. Nanoscale Nuclear Magnetic Resonance with a 1.9-Nm-Deep Nitrogen-Vacancy Sensor. 2014.

(9) Perona Martínez, F.; Nusantara, A. C.; Chipaux, M.; Padamati, S. K.; Schirhagl, R. Nanodiamond Relaxometry-Based Detection of Free-Radical Species When Produced in Chemical Reactions in Biologically Relevant Conditions. 2023, 30, 38. https://doi.org/10.1021/acssensors.0c01037.

(10) Vedelaar, T. A.; Hamoh, T. H.; Martinez, F. P. P.; Chipaux, M.; Schirhagl, R. Optimizing Data Processing for Nanodiamond Based Relaxometry. Adv Quantum Technol 2023. https://doi.org/10.1002/QUTE.202300109.
